# Differential partitioning of Gαi1 with the cellular microtubules: a possible mechanism of development of Taxol resistance in human ovarian carcinoma cells

**DOI:** 10.1186/1750-2187-1-3

**Published:** 2006-11-10

**Authors:** Hemant K Parekh, Mahesha Adikari, Bharathi Vennapusa

**Affiliations:** 1 Department of Pathology and Laboratory Medicine, Temple University School of Medicine, 3400 N. Broad Street, Philadelphia, PA 19140, USA

## Abstract

**Background:**

Taxol binds to the cellular microtubules and suppresses their dynamic instability. Development of tumor cell resistance to taxol is typically associated with increased expression of the drug efflux pump P-glycoprotein and/or alterations in the microtubules. Recently, changes in the dynamic instability of the microtubules have also been associated with development of taxol resistance in a lung cancer cell line. We have established a 250-fold taxol-resistant human ovarian carcinoma subline (2008/13/4) that does not display the typical alterations associated with development of drug resistance.

**Results:**

Utilizing the mRNA differential display technique, we observed increased expression of an alpha subunit of the guanine nucleotide-binding protein, Gαi1, in the taxol-resistant human ovarian carcinoma cell lines compared to the parental 2008 cells. Several isoforms of the α-subunit of the G protein have been identified and the Gαi (inhibitory) are so named because they inhibit the activity of adenylate cyclase leading to inactivation of the cAMP-dependent protein kinase A (PKA) pathway. In addition, Gαi1 is also known to bind to microtubules and activates their GTPase activity and thus induces depolymerization of the microtubules. In the present study we demonstrate that the intracellular level of cAMP and the PKA activity were higher in the taxol-resistant 2008/13/4 and the 2008/17/4 cells despite the increased expression of Gαi1 in these cells. Moreover, Gαi1 was found to be localized not on the cell membrane, but in intracellular compartments in both the taxol-sensitive and -resistant human ovarian carcinoma cells. Interestingly, increased association of the Gαi1 protein and the microtubules in the taxol-resistant cells compared to the parental 2008 cells was observed, both prior to and after treatment of these cells with taxol.

**Conclusion:**

Based on the opposing effects of taxol and the Gαi1 protein on the microtubule dynamic instability (taxol suppresses microtubule dynamic instability whilst the Gαi1 protein inhibits the suppression) our results indicate the operation of a novel pathway that would enable the cells to escape the cytotoxic effects of taxol.

## Background

The antimitotic anticancer drug paclitaxel (taxol) has a unique mechanism of action: unlike other mitotic spindle poisons (*viz*. vinca alkaloids), taxol binds to the N-terminal end of β-tubulin and promotes microtubule assembly [1-3]. The effect of taxol on the microtubule dynamics has been studied extensively in cell free systems as well in living cells [4-6]. Observations thus made indicate taxol-induced suppression of microtubule dynamics inhibits the rate and extent of shortening of microtubules causing aberrant mitotic spindles formation and cell cycle arrest [4-6].

Overexpression of P-glycoprotein (P-gp; which functions as a drug efflux pump, [7,8]) is the common phenotype observed in taxol-resistant tumor cells. In addition, considering its intracellular target, it is not surprising that resistance to taxol has also been associated with alterations in microtubules resulting in reduced drug-binding affinity or decreased intracellular levels of polymerized tubulin and/or changes in expression of specific tubulin isotypes [8-10]. Recently, Goncalves *et al*. reported association of increased microtubule dynamics with taxol resistance in lung cancer cells [11]. However, they did not identify the cellular mediators of the increased dynamic behavior of microtubule in the taxol-resistant cells.

We have established a series of taxol-resistant human ovarian adenocarcinoma cell lines [12]. The > 1500-fold taxol-resistant 2008/17/4 cell line displayed the classical multidrug resistance phenotype with overexpression of P-gp and cross-resistance to other natural product drugs [12]. In contrast, the 2008/13/4 cell line, which was 252-fold resistant to taxol relative to the parental cell line (2008), did not exhibit overexpression of the P-gp and/or alterations in the microtubules [12]. The intracellular accumulation of taxol in the 2008/13/4 cells was similar to that observed in 2008 cells. The *in vitro *binding of taxol to microtubules semi-purified from the 2008/13/4 and 2008 cells was also identical [12]. The basal levels of various α- and β-tubulin isotypes (except Bα-1) as well as polymerized tubulin were similar in the taxol-resistant and the parental cells (unpublished observation). This suggests that mechanisms not hitherto identified and not typically associated with development of taxol-resistance were operative in the 2008/13/4 sub-line. Considering the potential utility of taxol in the treatment of primary and cisplatin-resistant ovarian cancers, it was of great clinical importance to elucidate these mechanisms. Employing the mRNA differential display analysis we identified an increased expression of a protein involved in the G-protein coupled signal transduction pathway, named Gαi1, in the taxol-resistant cells compared to the parental cell.

The Gα proteins, based on sequence comparisons are subdivided into four classes, Gs, Gi, Gq and G12-13. The Gαi is the regulatory signaling molecule that **inhibits **the adenylate cyclase activity [13]. Increased expression of Gαi1 could potentially inhibit the adenylate cyclase activity and thus the cAMP mediated signal transduction (that act through the PKA). Decrease in the PKA activity leads to activation of the mitogen-activated protein kinase (MAPK) pathway initiated by c-raf-1 [14]. In resting cells, the Gαi forms a heterotrimeric complex (with the β- and γ- subunits of guanine nucleotide binding protein) that provides signal coupling through a series of G-protein coupled receptors [13]. Upon activation by an appropriate signal, the receptor interacts with the plasma membrane bound heterotrimeric G-protein complex and catalyzes the exchange of bound GDP for GTP in the α-subunit. Subsequently, the GTP-bound Gα subunit and the βγ-subunit dissociate from the receptor as well as from each other. The "active" α-subunit and the "free" βγ-subunit initiate cellular response by altering the activity of intracellular effector molecules [13].

However, recent studies have indicated that a large fraction of the Gαi proteins show perinuclear localization [15,16]. In addition, Roychowdhury *et al*. [17] have demonstrated that Gαi1 specifically binds to the microtubules and activates the GTPase activity of tubulin resulting in increased microtubule dynamic instability and increased depolymerization of the microtubules. We thus hypothesized that increased Gαi1 association with the microtubules in the taxol-resistant cells would be sufficient to inhibit the taxol-induced suppression of microtubule dynamic instability such that it would allow the taxol-bound microtubules to depolymerize. This would negatively influence the cytotoxic effects of taxol (on the formation of stable microtubules leading to mitotic arrest) and provide a mechanism *via *which drug-resistant cells would be able to evade the cytotoxic effects of taxol. Based on our results presented in this study as well as those of Roychowdhury *et al*. [17] and Goncalves *et al*. [11], it is conceivable that increased binding of Gαi1 with the microtubules in the drug-resistant cells could inhibit taxol-induced suppression of microtubule dynamics, thus allowing cells to complete mitosis.

## Results

Two distinct taxol-resistant sublines were utilized in this study. The 2008/17/4 subline displays a classical MDR phenotype; an increased expression of the P-gp and decreased intracellular taxol accumulation [12]. In contrast, the 2008/13/4 subline has negligible P-gp expression and no defect in the intracellular accumulation of taxol [12]. The basal levels of tubulin expression were similar in the parental 2008 and the taxol-resistant 2008/13/4 cells and the *in vitro *binding affinities of taxol to purified microtubules derived from these cells were not significantly different [12].

### Differential expression of Gαi1 in the taxol-resistant cells

**Figure 1:**
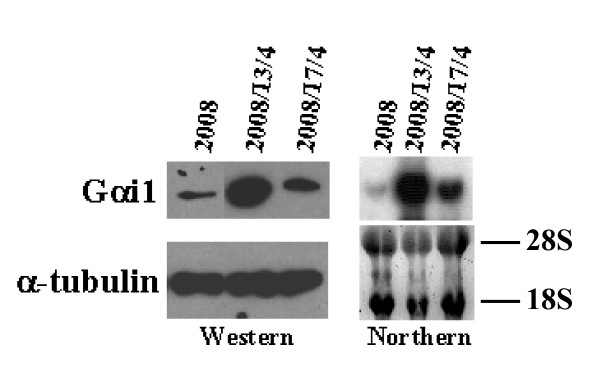
**Expression of Gαi1 in the taxol-sensitive and -resistant cells**. Whole cell lysate was prepared from each of the cell line by scraping into a buffer containing 20 mM Tris-HCl, pH 7.5, 150 mM NaCl, 1 mM EDTA, 1 mM EGTA, 1% (v/v) Triton-X-100, 0.5% (v/v) Nonidet P40, 2.5 mM Na pyrophosphate, 1 mM NaOV, 50 mM NaF and 1× protease inhibitor cocktail and incubated on ice for 15 minutes. The lysate was then centrifuged at 13,000 × *g *for 20 min and the supernatant was transferred to a fresh tube and stored at -80°C until use. Proteins (25 μg/lane) were separated on a SDS-PAG and transferred to a PVDF membrane. Western blotting analysis was performed using rabbit polyclonal antibody against Gαi1 and enhanced chemiluminescence reagents. Expression of α-tubulin was evaluated in the same lysates to ensure equal protein concentrations in each sample. For Northern blotting analysis, total RNA (20 μg) extracted from each cell was separated and transferred to Nylon membrane. Full-length Gαi1 cDNA was used as probe. The ethidium bromide stained RNA gel is shown in the bottom right-hand corner to ensure equal RNA loading.

To identify genes whose expression is either induced or reduced in the taxol-resistant cells, we systematically compared mRNA display patterns between parental (2008) and the taxol-resistant (2008/13/4 and 2008/17/4) cells. We choose only those bands that showed a consistent differential expression in the duplicate samples from the 2008/13/4 cells and/or 2008/17/4 cells compared to the 2008 cells. Furthermore, we chose only those bands that exhibited at least 3-fold change in densitometric intensity. We thus identified the upregulation of the alpha-i1 subunit of the guanine nucleotide binding protein mRNA (Gαi1; data not shown) in the 2008/13/4 and 2008/17/4 cells compared to the 2008 cells. The increased expression of the Gαi1 in the taxol resistant cells was confirmed by Northern and Western blotting analysis. As shown in Figure 1, a 15-fold and 6-fold higher expression of Gαi1 (protein and mRNA, respectively) was observed in the 2008/13/4 and 2008/17/4 cells, respectively, compared to the 2008 cells.

### The effect of taxol on the cAMP levels and PKA activity in the parental and taxol-resistant cells

The Gαi is a regulatory signaling molecule that **inhibits **the adenylate cyclase activity [13]. Increased expression of Gαi1 could potentially inhibit the adenylate cyclase activity and thus the cAMP mediated signal transduction (that act through the PKA). We assessed the basal levels of cAMP as well as the basal PKA activity in the parental and taxol-resistant cells. Effect of taxol exposure on the intracellular levels of cAMP and the PKA activity were also evaluated in these cells.

**Figure 2:**
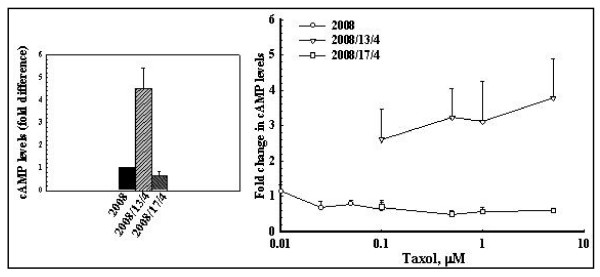
**Intracellular cAMP levels in the parental and taxol resistant cells before and after treatment with taxol**. cAMP levels were determined using an acetylated version of a cAMP measurement kit from Biomol Research Laboratories. A standard curve of known concentrations of acetylated cAMP was generated for each experiment. Untreated and taxol treated (16 hr) cells were processed as described (see Materials and Methods section). The cAMP level in the taxol resistant cells was compared with those observed in the 2008 cells (5.6 ± 1.1 pmol/mg protein; mean of 5 experiments each performed in duplicate) considered as 1.

The basal level of cAMP in the 2008, 2008/13/4 and 2008/17/4 cells was measured using a competitive immunoassay kit from Biomol Research Laboratories (Plymouth Meeting, PA). Surprisingly, the basal level of cAMP was 4.5-fold and 9-fold higher in the 2008/13/4 cells compared to the 2008 and 2008/17/4 cells, respectively (Fig. 2). These results indicate that increased expression of Gαi1 observed in the 2008/13/4 cells does not inhibit the basal AC activity in these cells. In contrast, treatment (for 16 hr) with increasing concentration of taxol did not alter the cAMP levels in the 2008, 2008/13/4 and 2008/17/4 cells significantly. It is likely that exposure to taxol causes a mild inhibition of adenylate cyclase activity with the resultant insignificant decrease in the intracellular levels of camp observed (Fig. 2).

**Figure 3:**
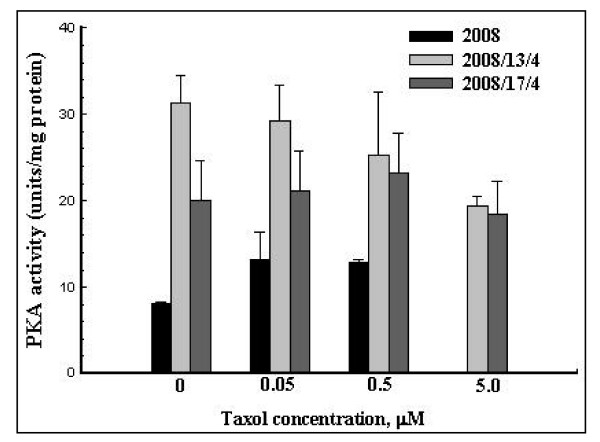
**PKA activity in the parental and taxol resistant cells before and after treatment with taxol**. PKA activity was determined using the Pierce Colorimetric Assay Kit that utilizes a fluorescent-labeled Kempeptide (a PKA-specific peptide (LRRASLG) substrate). Untreated and taxol treated cells were homogenized in a Dounce homogenizer (10 strokes with a tight fitting pestle) in a buffer containing 25 mM Tris-HCl, pH 7.4, 0.5 mM EDTA, 0.5 mM EGTA, 10 mM DTT and protease inhibitor cocktail. The homogenate was centrifuged at 13,000 × *g *for 20 min and the PKA activity was measured (in triplicate) in the supernatant fraction. Values shown are mean ± SD of 3 separate experiments.

We then determined the activity of cAMP-dependent Protein kinase A in the parental and taxol-resistant cells. As shown in Fig. 3, the PKA activity in the untreated 2008 cells was 8 units/mg protein and it was 4-fold (32 units/mg protein) and 2.5-fold (21 units/mg protein) higher in the 2008/13/4 and the 2008/17/4 cells, respectively. Treatment (for 16 hr) with increasing concentrations of taxol did not significantly affect the PKA activity in any of the cell lines studied (Fig. 3).

Based on these observations we concluded that overexpression of Gαi1 in the 2008/13/4 and 2008/17/4 cells does not inhibit the cAMP-dependent signal transduction pathways, otherwise inhibition of adenylate cyclase (due to increased activity of Gαi1) would have led to a decrease in the intracellular levels of cAMP and a decrease in the activity of the cAMP-dependent PKA.

### Association of Gαi1 with the microtubules in taxol resistant cells

Results presented thus far indicate that the mode of action of Gαi1 in the taxol-resistant cells maybe distinct from its ability to inactivate AC. Recent studies have demonstrated that Gαi1 specifically binds to the microtubules and activates the GTPase activity of tubulin [17]. Considering that the cellular target of taxol is the microtubule, we wanted to investigate whether alterations in the interaction of Gαi1 with the microtubules accompanied development of taxol resistance in the 2008/13/4 and the 2008/17/4 cells.

**Figure 4:**
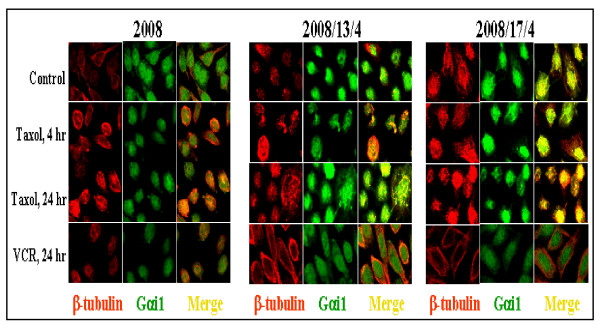
**Immunolocalization of Gαi1 in the 2008, 2008/13/4 and 2008/17/4 cells, before and after treatment with taxol**. To immunolocalize the Gαi1 protein, the 2008, 2008/13/4 and 2008/17/4 cells were grown on tissue culture-treated slides. After 48 hr, cells were treated with either taxol or vincristine (50 nM in case of 2008 cells, and 5 μM in case of the 2008/13/4 and 2008/17/4 cells) for 4 hr and 24 hr. At the end of each time period, the cells were fixed with 4% (w/v) paraformaldehyde and processed for immunostaining. After blocking nonspecific binding sites by incubating the slides with 5% (v/v) normal goat serum, the slides were incubated with primary antibody directed against Gαi1 (1:100 rabbit polyclonal) and β-tubulin (1:200 mouse monoclonal) for 1 hr. After washing in chilled PBS (3×), the slides were incubated with FITC-conjugated anti-rabbit antibody or rhodamine-conjugated anti-mouse antibody for 30 min. At the end of the incubation, the slides were washed again in PBS and mounted in media containing anti-fade. Localization of Gαi1 and β-tubulin was accomplished using an Olympus Confocal microscope.

**Figure 5:**
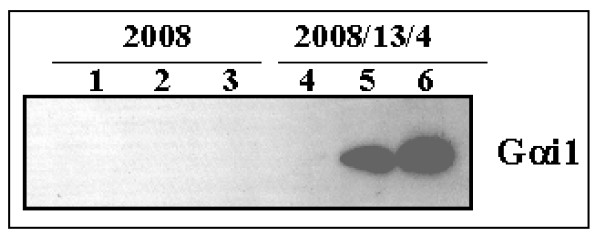
**Taxol dose-dependent increase in the association of Gαi1 protein with the microtubules exclusively in the 2008/13/4 cells**. The 2008 and 2008/13/4 cells were seeded at a density of 1 × 10^6^/ml and incubated under normal growth conditions for 36 hours. Thereafter, the 2008 cells untreated (lane 1) or treated for 24 hr with 25 nM (lane 2) and 500 nM (lane 3) taxol and the 2008/13/4 cells untreated (lane 4) or treated for 24 hr with 500 nM (lane 5) and 5 μM (lane 6) and then washed with chilled PBS (3 ×). The cytoskeletal fraction was isolated essentially as described previously [22]. Briefly, the attached cells were incubated in a microtubule-stabilizing buffer (0.1 M PIPES, 1 mM EGTA, 1 mM MgSO_4_, 2 M glycerol, pH 8.0) for 20 min. Thereafter, the cytosolic protein were removed by incubating the cells in the microtubule stabilizing buffer containing 0.1% NP-40 and protease inhibitor cocktail for 20 min. The cytoskeletal fraction (attached to the plastic dishes) was scraped into RIPA buffer (PBS containing 1% NP-40, 0.5% sodium deoxycholate and 0.1% SDS) containing protease inhibitor cocktail. The cytoskeletal fraction (5 μg protein/lane) was then subjected to SDS-PAGE. The separated proteins were transferred to PVDF membranes and Western blotting was performed using the polyclonal antibody against Gαi1.

**Figure 6:**
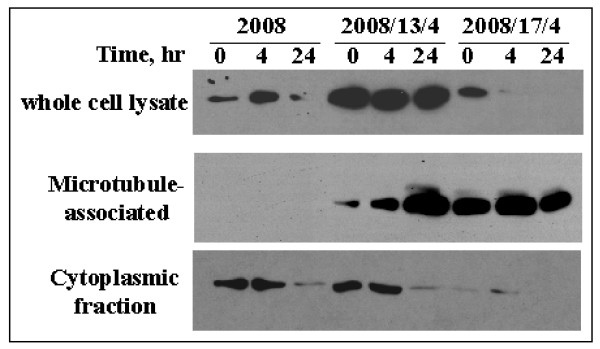
**Differential partitioning of Gαi1 to the microtubule fraction in the taxol-resistant cells**. The 2008, 2008/13/4 and 2008/17/4 cells were seeded at a density of 1 × 10^6 ^cells/plate and allowed to incubate in complete growth media for 36 hours. The cells were then treated with taxol (500 nM) for the indicated time. At the end of each time period, cells were processed for extraction of whole cell lysates (as described in Fig. 1), cytoskeletal fractions (as described in Fig. 5), cytoplasmic and nuclear fraction (utilizing the protocol supplied by manufacturer of the NE-PER extraction kit) and membrane fraction (utilizing the single step Mem-PER extraction kit). Protein (5 – 20 μg/lane) from each fraction were resolved on a 12% (w/v) SDS-PAG and then transferred to a PVDF membrane. Western blotting was performed utilizing the Gαi1 polyclonal antibody.

**Figure 7:**
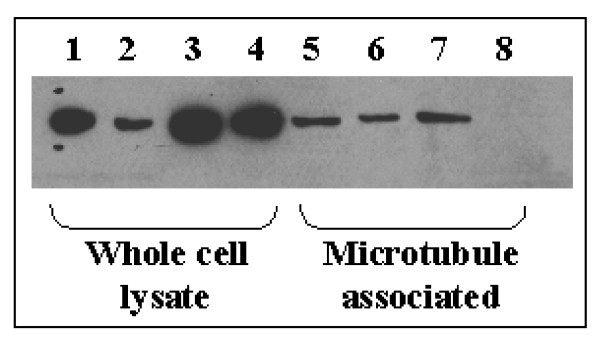
**Effect of Vincristine on the levels of Gαi1 in the whole cell lysate and cytoskeletal fraction from 2008 and 2008/13/4 cells**. The protein lysate (30 μg/lane) from untreated (lane 1,3,5,7) and vincristine treated (lane 2, 4, 6, 8) 2008 (lanes 1,2,5,6) and 2008/13/4 (lane 3,4,7,8) cells were separated by SDS-PAGE and then transferred to PVDF membrane. Presence of Gαi1 was assessed as described in Fig. 1.

Utilizing confocal microscopy (Fig. 4) and differential extraction (Figs. 5, 6), we now show an increased association of Gαi1 with the microtubules in the taxol-resistant cells compared to the sensitive cells, both prior to and after treatment with taxol. It is noteworthy here that vincristine, an anticancer drug known to depolymerize microtubules did not increase the association of Gαi1 with the microtubules in either the 2008 or the 2008/13/4 and 2008/17/4 cells (Figs. 4 and 7).

As shown in Fig. 4, a distinctly cytoplasmic localization of the Gαi1 protein (green) was observed in all the cell lines studied. The microtubules (red) were also stained with antibodies directed against β-tubulin. Co-localization of Gαi1 with microtubules (assessed by presence of yellow coloration) was not observed in the untreated 2008 cells, but was clearly visible in the taxol-resistant 2008/13/4 and 2008/17/4 cells. Moreover, treatment with taxol induced a time-dependent increase in the association of Gαi1 protein and the microtubule network in the taxol-resistant cells. This phenomenon was completely absent in the parental 2008 cells at 4 and 24 hr post-treatment with taxol. However, after 24 hr exposure to taxol, a few 2008 cells (<1%) displayed spotty co-localization of the Gαi1 protein with the microtubules. We also evaluated the effects of vincristine on the distribution of cellular Gαi1 protein. As shown in Fig. 4, treatment with vincristine decreased the association of the Gαi1 protein with the microtubules in the taxol-resistant cells without affecting its localization in 2008 cells. These results clearly illustrate that Gαi1 is differentially associated with the microtubules in the taxol-resistant cells in response to taxol.

In addition to the *in-situ *localization of the Gαi1 with the microtubules we also evaluated the association of Gαi1 with the microtubules and the effect of taxol treatment on this association in the 2008 and the 2008/13/4 cells utilizing a differential extraction procedure. As shown in Fig. 5, utilizing 5 μg protein/lane, Gαi1 was not found to be associated with the cytoskeletal fraction in the untreated 2008 cells or the 2008/13/4 cells. However, it should be pointed out that increasing the protein concentration to 30 μg does demonstrate a low basal level of Gαi1 bound to the cytoskeletal fraction in the 2008 and the 2008/13/4 cells (as shown in Fig. 7). Treatment of 2008 cells with 25 nM (IC_50_) or 500 nM (20 × IC_50_) concentration of taxol did not induce binding of Gαi1 with the microtubules. In marked contrast, treatment of the 2008/13/4 cells with 500 nM or 5 μM (IC_50_) concentration of taxol demonstrated a dose-dependent increase in the presence of Gαi1 in the cytoskeletal fraction.

Next, we evaluated the distribution of Gαi1 in the various cellular fractions (cytoplasmic, cytoskeletal, membrane and nuclear) before and after treatment with taxol in the 2008, 2008/13/4 and 2008/17/4 cells. As shown in Fig. 6, expression of Gαi1 was significantly higher in the whole cell lysates of untreated 2008/13/4 and 2008/17/4 cells compared to 2008 cells. Treatment with taxol did not affect the levels of Gαi1 in the 2008/13/4 whole cell lysate, however a significant decrease in Gαi1 levels was observed in the whole cell lysate obtained from 2008 and 2008/17/4 cells treated with taxol for 24 hr. Very low to negligible levels of Gαi1 were observed in the membrane fraction from each cell line (data not shown) and treatment with taxol did not increase the Gαi1 levels in the membrane fraction. Localization of Gαi1 in the nuclear fraction was not observed in any cell lines (data not shown). Similar to results obtained earlier (Fig. 5), association of Gαi1 with the microtubules was not observed at any time (before or after taxol treatment) in the 2008 cells. In contrast, a time-dependent increase in the partitioning of Gαi1 to the cytoskeletal fractions was observed in the 2008/13/4 cells. In case of the 2008/17/4 cells, a significant fraction of Gαi1 was already associated with the microtubules in the untreated cells, which was not affected by taxol treatment. When we evaluated the localization of Gαi1 in the cytoplasmic fraction in untreated and taxol- treated cells, we observed that levels of soluble Gαi1 in untreated 2008 and 2008/13/4 cells were similar, while very low levels of soluble Gαi1 were observed in the 2008/17/4 cells. The latter could be due to increased association of Gαi1 and microtubules in the untreated 2008/17/4 cells. Treatment with taxol for 24 hr decreased the levels of soluble Gαi1 in all the cell lines. Based on these observations and those reported in Fig. 4 (*in-situ *immunolocalization using confocal microscopy) we hypothesize that while decrease in the levels of cytoplasmic Gαi1 in the 2008 cells could be a direct result of increased degradation of the protein, decrease in the soluble Gαi1 in response to taxol in the 2008/13/4 cells and possibly 2008/17/4 cells is due to an increased partitioning of the Gαi1 from the cytoplasmic fraction to the cytoskeletal fraction.

We next determined whether the differential partitioning of Gαi1 to the microtubule fraction in the taxol-resistant cells was drug-specific (Figure 7). We treated the parental and taxol-resistant (2008/13/4) cells with vincristine and assessed the levels of Gαi1 in the whole cell lysate and in the cytoskeletal fraction. Vincristine was utilized because it is (like taxol) an antimitotic anticancer drug, however unlike taxol, vincristine induces depolymerization of the microtubules. Vincristine (50 nM) decreased the level of Gαi1 in the 2008 whole cell lysate (Figure 7, lane 2) without affecting the total levels in the 2008/13/4 cells (Figure 7, lane 4). Further, in contrast to the observation made with taxol, treatment of 2008 (50 nM) and 2008/13/4 (5 μM) cells with vincristine decreased the association of Gαi1 with the cytoskeletal fraction (Figure 7, lanes 6 and 8, respectively). These results suggest that the differential partitioning of Gαi1 to the microtubule fraction observed in the taxol-treated drug-resistant cells is indeed drug-specific providing further evidence that Gαi1 may be involved in inhibiting the taxol-induced suppression of microtubule dynamics.

## Discussion

Heterotrimeric G proteins transduce signals by virtue of their localization in close juxtaposition with the G-protein coupled receptors on the cytoplasmic side of the plasma membrane [13]. Activated receptors induce exchange of GDP for GTP on the Gα subunit and thus mediate the dissociation of Gα and βγ-subunits [13]. Dissociated Gαi1 decreases cAMP levels (due to inhibition of adenylate cyclase) and thus suppresses the cAMP-dependent PKA pathway. However, in the present study despite increased expression of Gαi1, under basal conditions and in the presence of taxol, intracellular cAMP levels as well as the PKA activity was higher in the taxol-resistant cells (Figs. 2 and 3) compared to the parental cells and >95% of the Gαi1 displayed intracellular localization (either in the cytoplasmic and/or in the cytoskeletal fraction; Fig. 4, 5, 6) in the parental and taxol-resistant cells. Furthermore, treatment with taxol induced a differential partitioning of Gαi1 in the cytoskeletal fraction specifically in the 2008/13/4 and 2008/17/4 cells.

These results suggest that the biochemical machinery necessary for targeting of Gαi1 is not operative under basal conditions or under conditions of stress associated with exposure to taxol either in the parental or in the taxol-resistant cells. Several studies indicate that covalent modification, including myristoylation and palmitoylation of Gα subunits are important in its targeting to the membrane [18,19]. Whilst constitutively activated mutants of Gα subunit have been identified, their importance in the cellular localization seems to be associated with the lack of affinity of these mutants with the βγ-subunit partner, an interaction necessary for membrane localization [20]. Thus, it is likely that alteration in the conformation of the Gαi1 protein due to posttranslational modifications and/or changes in the levels of expression of partner protein(s) (that aid in targeting it to the microtubules) is responsible for the differential localization of Gαi1 with the microtubules in the taxol-resistant cells. Cell membrane localization of Gαi1 is necessary for its inhibitory effect on the adenylate cyclase pathway. The absence of Gαi1 localization to the cell membrane in the taxol-sensitive and -resistant human ovarian cancer cell lines used in this study, could explain the lack of correlation between the increased intracellular levels of Gαi1 in the taxol-resistant cells with the cellular cAMP content and the activity of PKA in these cells.

Gαi1 is expressed in the 2008, albeit at levels lower than in the taxol-resistant cells; thus it is unclear as to why specific targeting of Gαi1 to the microtubules (before and after treatment with taxol) is observed only in the taxol-resistant cells. Whilst it is possible that differential posttranslational modification of the Gαi1 protein could account for its microtubule targeting in the taxol-resistant cells it is also likely that treatment of 2008/13/4 cells with taxol induces (a) the expression of a "chaperone" protein (bound to the microtubules and expressed only in the taxol-resistant cells) that could bind the soluble Gαi1 and thereby increase its association with the cytoskeleton or (b) binding of Gαi1 with a "chaperone" protein increases the ability of the complex to interact with the microtubules. While a third possibility exist; i.e., alteration in the microtubules themselves such that it increases the affinity of Gαi1 binding, such a change would lead to constitutively increased association of the Gαi1 with the microtubules, a phenomenon not observed in the 2008/13/4 cells.

We had originally hypothesized that the observed upregulation of the G-proteins might function *via *the adenylate cyclase – cAMP – PKA-mediated signal transduction pathway in the development of taxol resistance. In case of the 2008/13/4 cells, although changes were identified in this pathway these could not be directly attributed as an effect of increased expression of the Gαi1. These observations make a compelling argument that other subtle and uncommon effects of the Gαi1 may be involved in the development of taxol resistance observed in the 2008/13/4 and 2008/17/4 cells.

In addition to being membrane bound, several reports [15,16] including the present study have demonstrated that the Gαi1 protein is present in the perinuclear region of the cell and is probably involved in transfer of signals from cell surface receptors to intracellular effector molecules. Wang *et al*. recently demonstrated that the tubulin binds to purified Gαi1 and Gαs protein with high specificity but not to other members of the Gαi family of proteins [21]. Furthermore, Roychowdhury *et al*. demonstrated that binding of Gαi1 to tubulin dimers and/or microtubules resulted in activation of tubulin GTPase activity and modulation of microtubule polymerization dynamics [17]. An elegant model of regulation of microtubule dynamics in vivo by Gαi protein proposed by these authors suggest that binding of the G-protein to the end of a microtubule induces hydrolysis of GTP and subsequent loss of the stabilizing cap, resulting in microtubule depolymerization (a catastrophe event) [17]. In contrast, binding of taxol to microtubule leads to stabilization of microtubule polymer, in other words suppression of dynamic instability of microtubules (4–6). Taken together, it is very attractive to speculate that the increased expression of the Gαi1 protein would be able to induce depolymerization of the taxol-stabilized microtubule polymers in the taxol resistant 2008/13/4 and the 2008/17/4 cells, resulting in completion of mitosis and subsequent cell division.

## Conclusion

We hypothesize that all else being equal, the presence of increased quantity of Gαi1 protein in the vicinity of the taxol-bound microtubules is likely to negate the suppressive effects of the drug and allow microtubules to depolymerize at the end of mitosis and complete the cell cycle. The notion is that Gαi1 with its ability to activate the intrinsic tubulin-GTPase activity will enable taxol-stabilized microtubules to depolymerize. Studies are underway in this laboratory to ascertain whether such a phenomenon occurs in vitro utilizing microtubules purified from the taxol resistant cells.

## Methods

### Materials

Taxol was obtained from the Drug Synthesis and Chemistry Branch, Developmental Therapeutics Program, Division of Cancer Treatment, National Cancer Institute, National Institutes of Health. It was dissolved in DMSO at a final concentration of 20 mM and the concentration of the solvent never exceeded 0.1% in any experimental protocol. [α-^32^P] dCTP (3000 Ci/mmol) was purchased from Dupont-NEN Research Products. The random priming kit from Amersham Biosciences (Piscataway, NJ) was utilized for radioactively labeling the Gαi1 cDNA. The enhanced chemiluminescence reagents were from Pierce Biochemicals (Rockford, IL). The antibodies utilized in this study and their suppliers were; rabbit polyclonal Gαi1 antibody was from Santacruz Biotechnology Inc. (Santa Cruz, CA), mouse monoclonal β-tubulin antibody was from BD Biosciences (San Diego, CA), rhodamine-conjugated anti-mouse antibody and the FITC-conjugated anti-rabbit antibody was from Pierce Biochemicals (Rockford, IL).

### Cell lines

The parental human ovarian carcinoma cells, 2008 and its taxol-resistant derivatives (2008/13/4 and 2008/17/4) were maintained as described previously [12]. The 2008/13/4 and the 2008/17/4 cells were derived by exposure of the 2008 cells to stepwise increasing concentrations of taxol [12]. The 2008/13/4 cells are cross-resistant to other antimitotic drugs (*viz*., vincristine, vinblastine) and etoposide but are not cross-resistant to adriamycin and cisplatin. The 2008/13/4 cells have no defect in the transport of taxol, express negligible levels of P-glycoprotein and are 252-fold resistant to taxol compared to the 2008 cells. In contrast the > 1500-fold taxol-resistant 2008/17/4 cells display a classic multidrug resistant phenotype with cross-resistance to all natural product drugs tested, expression of high levels of P-glycoprotein and a significant defect in the intracellular accumulation of taxol. However, the binding of taxol to microtubules is similar in the taxol-resistant and the parental cells [12].

### Intracellular cAMP levels

The intracellular cAMP levels in the 2008, 2008/13/4 and 2008/17/4 cells were determined using an acetylated version of a cAMP measurement kit from Biomol Research Laboratories (Plymouth Meeting, PA). A standard curve of known concentrations of acetylated cAMP was generated for each experiment. The cAMP level (pmol/mg protein) in the taxol resistant cells was compared with those observed in the 2008 cells considered as 1.

### cAMP-dependent PKA activity

PKA activity was determined using the Pierce Colorimetric Assay Kit (Rockford, IL) that utilizes a fluorescent-labeled Kempeptide (a PKA-specific peptide (LRRASLG) substrate). The parental and taxol-resistant cells were seeded at a density of 2 × 10^6 ^cells/100 mm plate and incubated for 24 hr. Thereafter, the cells were either left untreated or treated with varying concentrations of taxol for the indicated time periods. Untreated and taxol treated cells were homogenized in a Dounce homogenizer (10 strokes with a tight fitting pestle) in a buffer containing 25 mM Tris-HCl, pH 7.4, 0.5 mM EDTA, 0.5 mM EGTA, 10 mM DTT and protease inhibitor cocktail. The homogenate was centrifuged at 13000 × *g *for 20 min and the PKA activity was measured (in triplicate) in the supernatant fraction as per the instructions of the kit supplier. Values shown are mean ± SD of 3 separate experiments.

### Protein preparations

The 2008, 2008/13/4 and 2008/17/4 cells were seeded at a density of 1 × 10^6^/ml and incubated under normal growth conditions for 36 hours. Thereafter, the cells were left untreated or treated with varying concentrations of taxol or vincristine for the indicated time periods. At the end of each time period the cells were washed with chilled PBS (3 ×) and various protein fractions were extracted as outlined below.

### Whole cell lysate

Whole cells lysate was prepared from each of the cell line by scraping cells into a buffer containing 20 mM Tris-HCl, pH 7.5, 150 mM NaCl, 1 mM EDTA, 1 mM EGTA, 1% (v/v) Triton-X-100, 0.5% (v/v) Nonidet P40, 2.5 mM sodium pyrophosphate, 1 mM sodium orthovanadate, 50 mM sodium fluoride and 1× protease inhibitor cocktail and incubating on ice for 15 minutes. Then, the lysate was centrifuged at 13,000 × *g *for 20 min and the supernatant was transferred to a fresh tube and stored at -80°C until use. Proteins were separated on a SDS-PAG and transferred to a PVDF membrane. Western blotting analysis was performed using rabbit polyclonal antibody against Gαi1 (Santacruz Biotechnology Inc., Santa Cruz, CA) and enhanced chemiluminescence reagents from Pierce Biochemicals (Rockford, IL).

### Cytoskeletal protein fraction

The cytoskeletal fraction was isolated essentially as described previously [22]. Briefly, the attached cells will be incubated in a microtubule-stabilizing buffer (0.1 M PIPES, 1 mM EGTA, 1 mM MgSO_4_, 2 M glycerol, pH 8.0) for 20 min. Thereafter, the cytosolic protein were removed by incubating the cells in the microtubule stabilizing buffer containing 0.1% NP-40 and protease inhibitor cocktail for 20 min. The cytoskeletal fraction (attached to the plastic dishes) was scraped into RIPA buffer (PBS containing 1% NP-40, 0.5% sodium deoxycholate and 0.1% SDS) containing protease inhibitor cocktail. This selective extraction procedure has been shown to be ideal for preserving the association between the cytoskeleton and other associated regulatory molecules [22]. The cytoskeletal fraction (5 μg protein/lane) was then subjected to SDS-PAGE. The separated proteins were transferred to PVDF membranes and Western blotting was performed using the polyclonal antibody against Gαi1 as described earlier.

### Subcellular protein fractions

The cells were processed for the extraction of cytoplasmic and nuclear fraction utilizing the protocol supplied by manufacturer of the NE-PER extraction kit (Pierce Biochemicals, Rockford, IL). The membrane fraction was extracted utilizing the single step Mem-PER extraction kit and protocol supplied by the manufacturer (Pierce Biochemicals, Rockford, IL). Protein (5 – 20 μg/lane) from each fraction were resolved on a 12% (w/v) SDS-PAG and then transferred to a PVDF membrane. Western blotting was performed utilizing the Gαi1 polyclonal antibody (Santacruz Biotech, Inc., Santacruz, CA) as described above.

### Northern blotting analysis

Total RNA (20 μg) extracted from each cell line was fractionated on 1% agarose-formaldehyde gel and then stained with ethidium bromide to ensure equal RNA loading. Thereafter the RNA was transferred to a Nylon membrane and probed with a full-length Gαi1 cDNA labeled with ^32^P-dCTP with a random priming kit from Amersham Biosciences (Piscataway, NJ). Hybridization was performed at 55°C overnight and nonspecific binding was removed by washing the nylon membrane in a high stringency buffer at 55°C for 30 min.

### Immunolocalization of Gαi1

To immunolocalize the Gαi1 protein, the 2008, 2008/13/4 and 2008/17/4 cells were grown on tissue culture-treated slides. After 48 hr, cells were treated with either taxol or vincristine (50 nM in case of 2008 cells, and 5 μM in case of the 2008/13/4 and 2008/17/4 cells) for 4 hr and 24 hr. At the end of each time period, the cells were rapidly rinsed in chilled PBS and then fixed with 4% (w/v) paraformaldehyde and processed for immunostaining. After blocking nonspecific binding sites by incubating the slides with 5% (v/v) normal goat serum, the slides were incubated with primary antibody directed against Gαi1 (1:100 rabbit polyclonal) and β-tubulin (1:200 mouse monoclonal) for 1 hr. After washing in chilled PBS (3×), the slides were incubated with FITC-conjugated anti-rabbit antibody or rhodamine-conjugated anti-mouse antibody for 30 min (Pierce Biochemicals, Rockford, IL). At the end of the incubation, the slides were washed again in PBS and mounted in media containing anti-fade. Localization of Gαi1 and β-tubulin was accomplished using an Olympus Confocal microscope.

## Abbreviations

Taxol, Paclitaxel; P-gp, P-glycoprotein; Gαi1, guanine nucleotide binding protein, subunit alpha i1; PKA, Protein kinase A; MAPK, Mitogen-activated protein kinase; PBS, phosphate-buffered saline; SDS-PAGE, sodium dodecyl sulfate-polyacrylamide gel electrophoresis; MDR, multidrug-resistance.

## Competing interests

The author(s) declare that they have no competing interests.
